# PW02-006 - PAPA syndrome clinical spectrum and IL1B release

**DOI:** 10.1186/1546-0096-11-S1-A146

**Published:** 2013-11-08

**Authors:** A Omenetti, R Caorsi, S Carta, L Delfino, A Martini, A Rubartelli, M Gattorno

**Affiliations:** 1 UO Pediatria II, G Gaslini IRCCS, , AOU San Martino - IST, Genoa, Italy; 2 University of Genoa, AOU San Martino - IST, Genoa, Italy; 3 Cell Biology, IRCCS, AOU San Martino - IST, Genoa, Italy

## Introduction

Pyogenic sterile Arthritis Pyoderma gangrenosum and Acne (PAPA) syndrome is a rare autosomal dominant inherited autoinflammatory disease caused by mutations in Proline-serine-threonine phosphatase-interacting protein 1 (PSTPIP1). In childhood, the syndrome is featured by recurrent sterile, erosive arthritis, potentially leading to joint destruction. By puberty, cutaneous symptoms become predominant, with recurrent onset of pathergy, abscesses, severe cystic acne, and pyoderma gangrenosum. Typically, both articular and cutaneous outcomes occur following a minor trauma. PSTPIP1 may interact with NLRP3 and caspase-1 but a clear involvement of IL-1β is still controversial. While anti-IL1 treatment seems to be effective on joint manifestations, IL inhibition does not display the same effectiveness in the management of skin lesions.

## Objectives

To investigate in our PAPA cohort whether 1) PSTPIP1 mutated monocytes display enhanced IL1β secretion; 2) different PSTPIP1 mutations and/or clinical manifestations and disease activity correlate with degree in IL1β pathway activation; 3) IL1β release is mediated by NLRP3.

## Methods

Fourteen PAPA patients were examined. Thirteen genetically confirmed patients (2 children and 11 adults) carrying different PSTPIP1 mutations (N=11 E250Q, N=1 E250K, N=1 E256G) and 1 pediatric patient genetically negative for common PSTPIPI1 variants, were analyzed and compared to 30 healthy donors (HD). Peripheral blood primary human monocytes were freshly isolated and studied at baseline and after 3-6-18 hours (h) of LPS-induced *in vitro* activation, and pattern of IL-1β secretion was assessed by ELISA. The involvement of NLRP3 was investigated by *in vitro* silencing.

## Results

Monocytes isolated from PAPA patients tend to secrete higher levels of IL1β but variability occur even in the presence of the same PSTPIP1 variant. IL1 β secretion is higher in patients displaying prevalent articular vs skin manifestations, and increases in the presence of acute phase reactants elevation and/or joint/skin lesions. The blockage of NLRP3 activity leads to IL1 release inhibition in both PAPA and HD monocytes.

## Conclusion

IL1 β secretion is higher in PAPA patients displaying prevalent articular vs skin manifestations, correlates with disease activity and is mediated by NLRP3.

## Disclosure of interest

None declared.

